# MT1G inhibits the growth and epithelial-mesenchymal transition of gastric cancer cells by regulating the PI3K/AKT signaling pathway

**DOI:** 10.1590/1678-4685-GMB-2021-0067

**Published:** 2022-02-11

**Authors:** Guofeng Xu, Linfeng Fan, Shufeng Zhao, Canhui OuYang

**Affiliations:** 1 First Affiliated Hospital of Gannan Medical University, Department of Gastroenterology, Ganzhou City, Jiangxi Province, China.; 2 First Affiliated Hospital of Gannan Medical University, Department of Gastrointestinal Surgery, Ganzhou City, Jiangxi Province, China.

**Keywords:** MT1G, GC, PI3K/AKT signaling pathway, cell growth, EMT

## Abstract

Gastric carcinoma (GC) is a malignant tumor that has high mortality and morbidity worldwide. Although many efforts have been focused on the development and progression of GC, the underlying functional regulatory mechanism of GC needs more clarification. Metallothionein 1G (MT1G) is a member of the metallothionein family (MTs), and hypermethylation of MT1G occurred in a variety of cancers, including gastric cancer. However, the functional mechanism of MT1G in GC remains unclear. Here, we demonstrated that MT1G was down-regulated in GC tissues and cells. Overexpression of MT1G inhibited cell proliferation, foci formation and cell invasion, while knockdown of MT1G increased cell proliferation, foci formation and cell invasion. In addition, MT1G overexpression inhibited cell cycle progression and MT1G deficiency exerted opposite phenotype. p-AKT was negatively regulated by MT1G. In summary, our study reveals that MT1G exerts crucial role in regulating of cell proliferation and migration of gastric cancer, providing new insights for MT1G-related pathogenesis and a basis for developing new strategies for treatment of GC.

## Introduction

Gastric cancer (GC) is a malignant tumor that originates from the epithelium of the gastric mucosa. It ranks first among various malignant tumors in China. Due to dietary changes, increased work pressure and *Helicobacter pylori* infection, the occurrence of GC appears to be younger. In the past, the only treatment for gastric cancer is surgical resection (Kai *et al*., 2018). However, the early symptoms of gastric cancer are difficult to detect, so majority of patients with gastric cancer are difficult to diagnose early, and therefore losing the chance of cure and resection. According to the classification of TCGA and ACRG, the microsatellite instability (MSI) group represents an important subset of GCs. Because it has better survival results than microsatellite stable tumors in the resectable stage, it has attracted many research attentions. Even now chemotherapy is also used as a clinical treatment method for gastric cancer, but GC has a strong ability to metastasize, so the prognosis of GC patients is still very poor (Peng *et al*., 2018). Therefore, studying the mechanism and process of GC can be better used for clinical treatment and diagnosis of GC (Waldum and Fossmark, 2018).

Metallothionein 1G (MT1G) is a member of the metallothionein family (MTs), a highly conserved low molecular weight protein rich in cysteine residues (Romero-Isart and Vasak, 2002; Si and Lang, 2018). Most of the biological functions of MTs are related to metal binding, including heavy metal detoxification, providing zinc/copper for enzymes and transcription factors, and preventing oxidative stress (Coyle *et al*., 2002; Vasak, 2005; Nielsen *et al*., 2007). Previous studies have shown that the expression of MT1G is suppressed due to promoter methylation. MT1G hypermethylation occurs in a variety of tumors, including hepatocellular carcinoma, colorectal cancer, prostate cancer and gastric cancer (Huang *et al*., 2003; Henrique *et al*., 2005; Arriaga *et al*., 2012). In thyroid cancer, the restoration of MT1G expression can inhibit the proliferation of thyroid cancer cells in vivo and in vitro, suggesting that it has a tumor suppressor effect (Ferrario *et al*., 2008). Further studies have found that MT1G inhibits thyroid cancer cells by inhibiting the proliferation and migration through the PI3K/AKT pathway (Fu *et al*., 2013). In addition, Wu etc. analyzed the DNA chip expression data of GEO database GSE26942, GSE33335, GSE63089 and GSE79973 and found that *MT1G* is low-expressed in gastric cancer (Wu *et al*., 2020). However, there are few reports about the role of MT1G in gastric cancer, and its mechanism is unclear.

There are several signaling pathways involved in gastric cancer development and progression, including Wnt/beta-catenin (Nunez *et al*., 2011), Hedgehog (Yao *et al*., 2019), TGF-β/SMAD3 (Li *et al*., 2021), MAPK (Zhang *et al*., 2014), and PI3K/AKT/mTOR (Chen *et al*., 2018; Wu *et al*., 2019) signaling pathway. Previous study reported that aberrant activation of Hedgehog signaling pathway results in pathological consequences in human gastric cancer. FoxC1 promoted the proliferation of GC cells by negatively regulating DKK1 expression, thus promoting the activation of Wnt pathway (Jiang *et al*., 2021). Li and his colleagues demonstrated that IGHG1 promotes the proliferation, migration and invasion of gastric cancer cells through regulation of TGF-β/SMAD3 signaling pathway (Li *et al*., 2021). HOXB13, as a tumor inducer, facilitated gastric cancer cell migration and invasion by upregulation of IGF-1R, thereby increasing the activation of PI3K/AKT/mTOR signaling pathway (Guo *et al*., 2021). Furthermore, previous study reported that MT1G acted as a tumor suppressor in thyroid cancer through regulating the PI3K/AKT signaling pathway. Therefore, we speculated that MT1G may functions as a similar suppressor in regulation of GC via PI3K/AKT signaling pathway.

In this study, we provided evidence that MT1G is down-regulated in GC cells and tissues. MT1G inhibits the growth and epithelial-mesenchymal transition of gastric cancer cells by regulating the PI3K/AKT signaling pathway. 

## Material and Methods

### Cell lines and cell culture

We used five types of cell lines in this study, including normal gastric mucosa cells: GES-1, and gastric cancer cells: NCI-N87, HGC-27, SNU-1 and HS-746T (All cell lines were uniformly validated by STR, pollution-free, and there was no mutation of genes, including *MT1G* in these cells). All cells were purchased from the Cell Bank of the Chinese Academy of Sciences (Shanghai, China) and cultured in Dulbecco’s modified Eagle’s medium (DMEM) supplemented with 10% FBS and 100 U/ml penicillin/streptomycin. All the cell lines were maintained in 37 ℃, 5% CO_2_ incubator. The experimental manipulations performed have been previously reported (Dong *et al*., 2020).

### RNA isolation and real-time quantitation PCR

Total RNA was extracted using TRIzol reagent (Ambion, CA, USA). A total of 1 µg of RNA was reverse-transcribed using the ImProm-IITM Reverse Transcription System (Promega, WI, USA). Quantitative real-time RT-PCR was conducted using SYBR GREEN qPCR Super Mix (Invitrogen, CA, USA). A standard amplification protocol was used according to the supplier’s directions. Primers were listed as following. 

*MT1G* forward: 5’- CTTCTCGCTTGGGAACTCTA -3’; 

*MT1G* reverse: 5’- AGGGGTCAAGATTGTAGCAAA -3’;

*GAPDH* forward: 5’- AGACAGCCGCATCTTCTTGT-3’; 

*GAPDH* reverse: 5’- CTTGCCGTGGGTAGAGTCAT-3’. 

### Western blotting analysis

Western blotting was performed as previously depicted (Dong *et al*., 2018). And immunoblotted with the following antibodies: anti-rabbit MT1G (1:1000, abcam, ab193329, England), anti-mouse E-cadherin (1:1000, Santa Cruz, sc-8426, USA), anti-mouse N-cadherin (1:1000, Santa Cruz, sc-8424, USA), anti-mouse snail (1:1000, Santa Cruz, sc-271977, USA), anti-mouse p-AKT (1:1000, Santa Cruz, sc-377556, USA), anti-mouse AKT (1:1000, Santa Cruz, sc-5298, USA), anti-mouse β-actin (1:1000, Santa Cruz, sc-8432, USA). Then, the PVDF membranes were washed and secondary antibodies were applied 1:5000 for 1 h at room temperature. The immunoreactions were visualized with chemiluminescent ECL reagent. Western blotting assays were performed according to a standard protocol and densitometry volume of the target bands was quantified using Fiji software.

### Cell proliferation and colony formation assays

The cell proliferation and cytotoxicity assay were performed as previously described (Yu *et al*., 2019). 1 × 10^4^ cells were seeded in a 96-well-plate in triplicate and the colorimetric CCK-8 Assay kit (keygenbio) was used to monitor the cell proliferation rate at 48h. For the clonogenic assay, 1 × 10^3^ cells were seeded in 6-well plates to form colonies. After 72 h, colonies were stained, photographed, and scored. Colonies with no fewer than 50 cells per colony were counted.

### Flow cytometry analysis

Cells were seeded into six-well plate before analysis, then collected, fixed, and incubated at -20 °C overnight. Cells were treated with 10 mg/ml RNase for 30 min and stained with 5 mg/ml of propidium iodide, and then subjected to cell cycle analysis by a flow cytometry (Becton Dickinson, CA).

### Transwell invasion assays

Cells were seed into upper chamber of Transwells (BD biosciences, San Jose, CA) in 24-well plates. And cells were serum-starved overnight in upper chamber and Dulbecco’s modified Eagle’s medium (DMEM) supplemented with 10% FBS and 100 U/ml penicillin/streptomycin was used as a chemoattractant in the lower chamber. 24 h later, cell attached to the membrane upper chamber were stained, photographed, and scored.

### Statistical analysis

Student’s *t* test and one-way ANOVA were used in statistical analyses. Data were presented as means ± SEM of three independent experiments. A P-value of 0.05 or less was considered to be statistically significant.

## Results

### MT1G was downregulated in gastric tissues and cells

**Figure 1 -:**
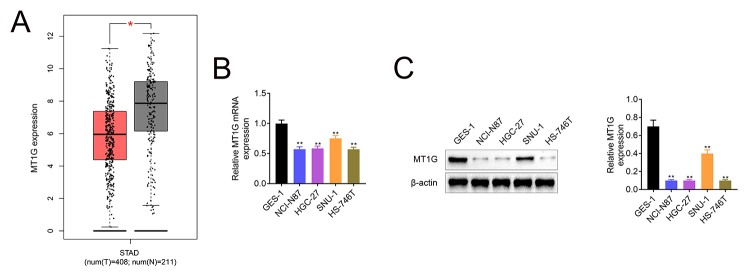
MT1G is low expression in gastric cancer tissues and cells. (A) The expression of MT1G in GC tissue and para-carcinoma normal tissue specimens was determined from TCGA analysis. (B&C) The expression of MT1G in GC cells was measured by RT-qPCR (B) and western blotting (C). Error bars represent data from three independent experiments (mean±SD). *P<0.05, **P<0.01.

MT1G, as a member of MTs family, has been revealed to exert crucial role in tumorigenesis, including gastric cancer. In order to clarify the mechanism of MT1G in GC, we analyzed its expression in tissues of GC (data from TCGA). As shown in Figure 1A, MT1G was downregulated in GC tissues compared with matched normal tissues. Next, we analyzed MT1G expression in cell lines by RT-qPCR (Figure 1B) and western blotting (Figure 1C). Compared with normal gastric cell GES-1, MT1G was low-expressed in gastric cancer cells (NCI-N87, HGC-27, SNU-1 and HS-746T). Collectively, these data suggested that MT1G is down-regulated in GC tissues and cell lines.

### MT1G negatively regulated cell proliferation and foci formation

**Figure 2 -:**
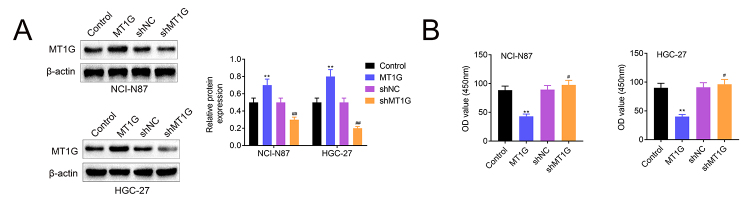
MT1G facilitates gastric cells growth. (A) NCI-N87 and HGC-27 cells were transfected with indicated plasmids, and the expression of MT1G was determined by western blotting. (B) The cell proliferation in MT1G overexpression or knockdown NCI-N87 and HGC-27 cells was tested by the CCK-8 assay. Error bars represent data from three independent experiments (mean±SD). **P<0.01, #P<0.05, ## P<0.01.

**Figure 3 -:**
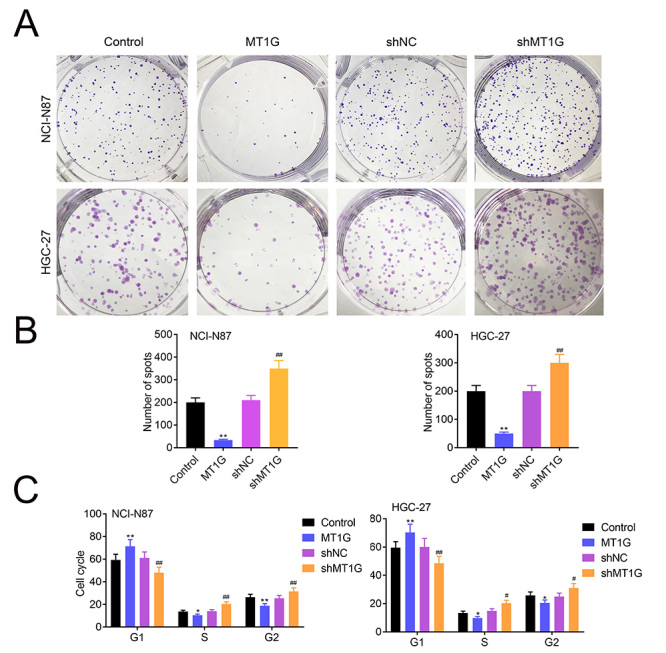
MT1G inhibits cell cycle progression of GC cells. (A and B) Clonogenic assay was performed to measure the capacity of foci formation in knockdown or overexpression of MT1G NCI-N87 and HGC-27 cells. (C) The effects of MT1G overexpression, knockdown on cell-cycle progression in NCI-N87 and HGC-27 cells were determined by propidium iodide staining and flow cytometry analysis. Error bars represent data from three independent experiments (mean±SD). *P<0.05, **P<0.01, #P<0.05, ## P<0.01.

Previous study demonstrated that MT1G functions as a tumor suppressor in thyroid cancer thus suppressing cell proliferation in thyroid cancer cells (Ferrario *et al*., 2008; Fu *et al*., 2013). We speculated that MT1G may have the same function in gastric cancer cells. To verify this speculation, we constructed stable cell lines for MT1G overexpression and knockdown in NCI-N87 and HGC-27 cells (Figure 2A). Then we analyzed the cell proliferation in GC cell lines. As shown in Figure 2B, overexpression of MT1G in NCI-N87 and HGC-27 significantly impeded cell proliferation compared with the control cells. In contrast, knockdown of MT1G remarkably increased cell proliferation (Figure 2B). In addition, reinforcing MT1G expression decreased colony formation while MT1G deficiency induced foci formation in NCI-N87 and HGC-27 cells (Figures 3A and 3B). Taken together, our data revealed that MT1G negatively regulated cell proliferation and foci formation in GC cells.

### MT1G inhibits cell cycle progression of gastric cancer

Next, we investigated whether MT1G regulates cell cycle progression in GC cells. We demonstrated that MT1G overexpression constrained cell cycle progression by increasing the percentage of cells in G1 phase and by decreasing the percentage of cells in S and G2 phase in NCI-N87 and HGC-27 cells (Figures 3C and S1). In contrast, MT1G knockdown restrained cell cycle progression by decreasing the percentage of cells in G1 phase and by increasing the percentage of cells in S and G2 phase NCI-N87 and HGC-27 (Figures 3C and S1). Taken together, our data demonstrated that MT1G negatively regulates cell cycle progression of GC cell lines.

### MT1G negatively regulates cell invasion

**Figure 4 -:**
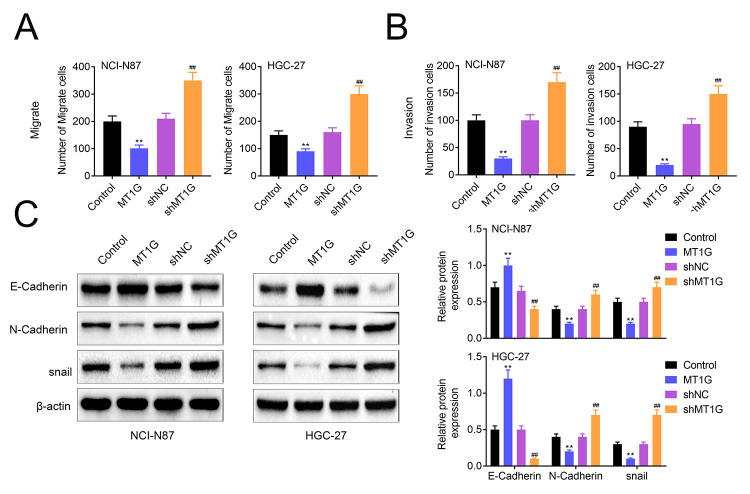
MT1G negatively regulates cell migration and invasion. (A and B) Transwell assay and transwell matrigel assay were used to measure cell migration and invasion in MT1G overexpression or knockdown NCI-N87 and HGC-27 cells. (C) Western blotting analysis EMT related proteins expression in knockdown or overexpression of CKS2 NCI-H2170 cells. MT1G overexpression or knockdown was shown in NCI-N87 and HGC-27 cells. Error bars represent data from three independent experiments (mean±SD). **P<0.01, ## P<0.01.

Over the past few years, it has been reported that MT1G deficiency promotes thyroid cancer cells proliferation and metastasis. Similarly, we measured cell invasion in GC cell by transwell assay, and as shown in Figures 4A and S2, MT1G overexpression constrained cell invasion in NCI-N87 and HGC-27 while MT1G deficiency exerted opposite effect. E-cadherin is a marker in regulation of EMT, which has important role in cell migration and invasion. Mechanically, E-cadherin was upregulated by MT1G overexpression and inhibited by MT1G inhibition. N-cadherin and snail were suppressed in MT1G upregulation NCI-N87 and HGC-27 cells, while increased in MT1G knockdown NCI-N87 and HGC-27 cell lines (Figures 4B and S2). Taken together, these data suggested that MT1G negatively regulates cell invasion via EMT signaling.

### MT1G deficiency induces PI3K/AKT signaling

**Figure 5 -:**
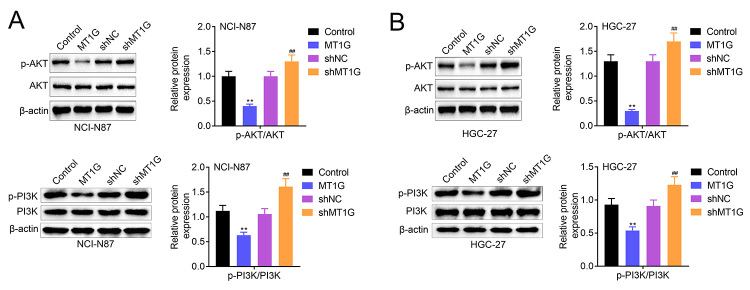
MT1G deficiency induces PI3K/AKT signaling in gastric cancer cell. (A-B) Gastric cancer cells (NCI-N87 and HGC-27) were divided into four groups based on its different treatment with plasmids transfection, including MT1G overexpression plasmids and related control plasmids, MT1G knockdown plasmids-sh*MT1G* and matched negative control plasmids-shNC. The expression of AKT, p-AKT, PI3K, p-PI3K in MT1G overexpression or knockdown NCI-N87 (A) and HGC-27 (B) cells was measured by western blotting. Error bars represent data from three independent experiments (mean±SD). **P<0.01. ##P<0.01.

**Figure 6 -:**
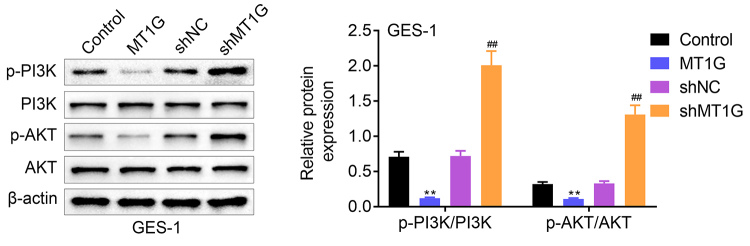
MT1G deficiency induces PI3K/AKT signaling in gastric normal cell. Gastric normal cells (GES-1) were divided into four groups based on its different treatment with plasmids transfection, including MT1G overexpression plasmids and related control plasmids, MT1G knockdown plasmids-sh*MT1G* and matched negative control plasmids-shNC. The expression of AKT, p-AKT, PI3K, p-PI3K in MT1G overexpression or knockdown GES-1 cells was measured by western blotting. Error bars represent data from three independent experiments (mean±SD). ＊＊P<0.01. ##P<0.01.

Next, we analyzed the effect of MT1G expression on regulating PI3K/AKT signaling pathway. Here, our results showed that p-AKT and p-PI3K were remarkably downregulated in MT1G overexpression NCI-N87, HGC-27 and GES-1 cells compared with the control group (Figures 5A and 5B and Figure 6). In contrast, p-AKT and p-PI3K were significantly upregulated in MT1G knockdown NCI-N87, HGC-27 and GES-1 cells (Figures 5A and 5B and Figure 6). Taken together, these data suggested that MT1G negatively regulates PI3K/AKT signaling pathway.

## Discussion

Gastric cancer is one of the most common malignant tumor, which accounts for 5.7% morbidity and 8.2% mortality in all kinds of cancer types all over the world (Bray *et al*., 2018). Based on its complex, histopathology, and clinical characters, such as drug resistance and lower survival rate, GC has a poor prognosis (Fattahi *et al*., 2020). Over the past twenty years, numerous studies have reported that the development and progression of gastric cancer involves diverse signaling pathways. However, the function mechanisms of GC development and progression are not fully clarified. 

*MT1G* is a DNA methylation-related gene which is reported to play an important role in various types of cancer (Henrique *et al*., 2005; Roth *et al*., 2006; Sakamoto *et al*., 2010; Arriaga *et al*., 2012; Fu *et al*., 2013; Zeng *et al*., 2018). In this study, we demonstrated for the first time that MT1G is down-regulated in GC cells and tissues compared with the control normal cells and tissues, respectively, suggesting that the low expression of MT1G may be benefit for GC development and progression. Its down-regulated expression in GC is in agreement with previous study which reported that MT1G decreased expression is significantly associated with HCC progression (Zeng *et al*., 2018). Furthermore, our results also indicated that MT1G negatively regulated cell proliferation, foci formation, cell cycle progression, cell invasion and epithelial-mesenchymal transition. Mechanically, MT1G mediated cell growth and cell cycle through PI3K/AKT signaling pathway. 

Previous study demonstrated that M1G exerts a tumor suppressor role in HCC and thyroid cancer (Huang *et al*., 2003; Ji *et al*., 2014). We revealed the similar inhibition function of MT1G in NCI-N87 and HGC-27 cells, which showed that MT1G negatively regulates tumorigenesis of GC. In addition, MT1G down-regulated expression in prostate cancer and hepatocellular carcinoma resulted from hypermethylation of its promoter. We speculated that the low expression of MT1G in gastric cancer maybe also related to the methylation modification, which needs further investigation. Functionally, MT1G exerted a suppressor role in regulating cell proliferation, colony formation and cell cycle progression. In addition, MT1G could also significantly mediates cell invasion, which was regulated by the epithelial-mesenchymal transition (EMT) process activation (Fu *et al*., 2013). Our data reported that MT1G remarkably decreased cell invasion through upregulation of E-cadherin expression and downregulation of N-cadherin and snail expression. Based on these, MT1G reversed the EMT process via E-cadherin upregulation.

EMT is one of the common features of cancers, which involves variety of signal pathways, including Wnt/beta-catenin, TGFbeta, and PI3K/AKT pathway (Chiu *et al*., 2019; Lambies *et al*., 2019; Zhang *et al*., 2020). For PI3K/AKT pathway, the level of p-AKT is a marker of pathway activation (Ge *et al*., 2018). In addition, the PI3K/AKT pathway was also positively associated with EMT process, and activation of the pathway facilitated cell proliferation, migration and invasion by GPER1 inhibition (Xu *et al*., 2020). We acquired the similar results in this study, and the results showed that MT1G negatively regulated p-AKT protein expression. Further study is needed to measure how MT1G regulates cell growth and EMT process via PI3K/AKT signal pathway in gastric cancer.

In conclusion, we demonstrated that MT1G inhibits cell proliferation, colony formation, cell invasion, cell cycle progression and EMT. We found that MT1G exerts a crucial role in the negative regulation of cell growth and invasion, probably mediated by the PI3K/AKT signaling pathway, although the molecular mechanisms still require further investigation. 

## Supplementary Material

The following online material is available for this article.

Figure S1 - MT1G inhibits cell cycle progression of GC cells.

Figure S2 - MT1G negatively regulates cell migration and invasion.
