# Supplementary material for: MT1G inhibits the growth and epithelial-mesenchymal transition of gastric cancer cells by regulating the PI3K/AKT signaling pathway
Source: Genet Mol Biol. 2022 Feb 11;45(1):e20210067. doi: 10.1590/1678-4685-GMB-2021-0067 (PMC8846298; doi:10.1590/1678-4685-GMB-2021-0067)
Supplement: Figure S1 - [file 1415-4757-GMB-45-1-e20210067-s1.pdf]

https://doi.org/10.1590/1678-4685-GMB-2021-0067

# Supplementary Material to "MT1G inhibits the growth and epithelial-mesenchymal transition of gastric cancer cells by regulating the PI3K/AKT signaling pathway"

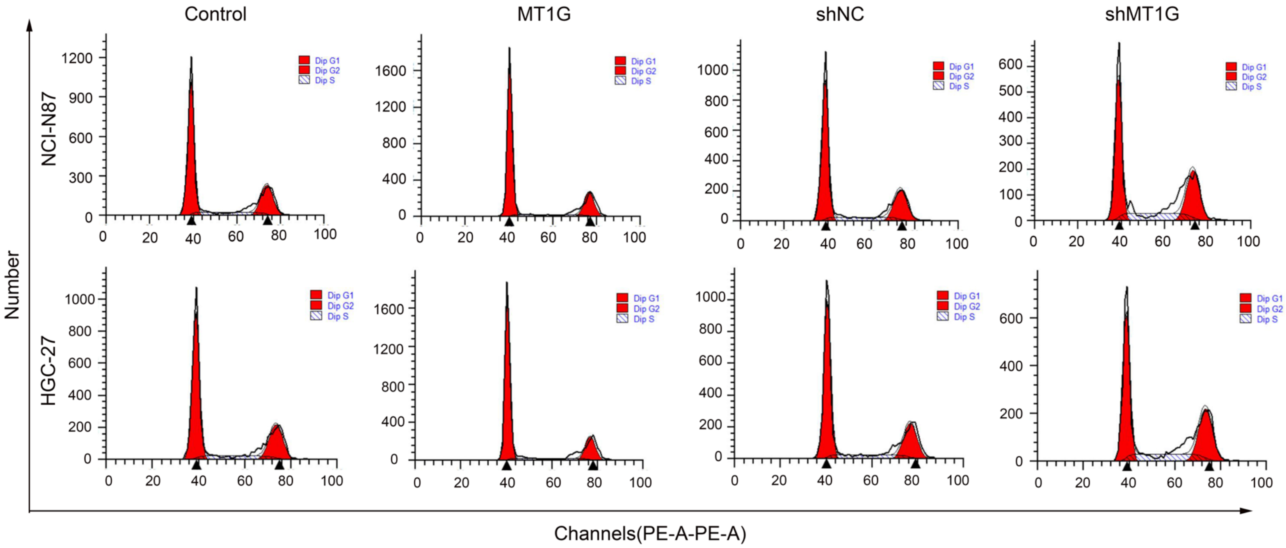


**Figure S1** - MT1G inhibits cell cycle progression of GC cells. The effects of MT1G overexpression, knockdown on cell-cycle progression in NCI-N87 and HGC-27 cells were determined by propidium iodide staining and flow cytometry analysis.
