# Supplementary material for: MT1G inhibits the growth and epithelial-mesenchymal transition of gastric cancer cells by regulating the PI3K/AKT signaling pathway
Source: Genet Mol Biol. 2022 Feb 11;45(1):e20210067. doi: 10.1590/1678-4685-GMB-2021-0067 (PMC8846298; doi:10.1590/1678-4685-GMB-2021-0067)
Supplement: Figure S2 - [file 1415-4757-GMB-45-1-e20210067-s2.pdf]

Genetics and Molecular Biology

https://doi.org/10.1590/1678-4685-GMB-2021-0067

Supplementary Material to "MT1G inhibits the growth and epithelial-mesenchymal transition of gastric cancer cells by regulating the PI3K/AKT signaling pathway"

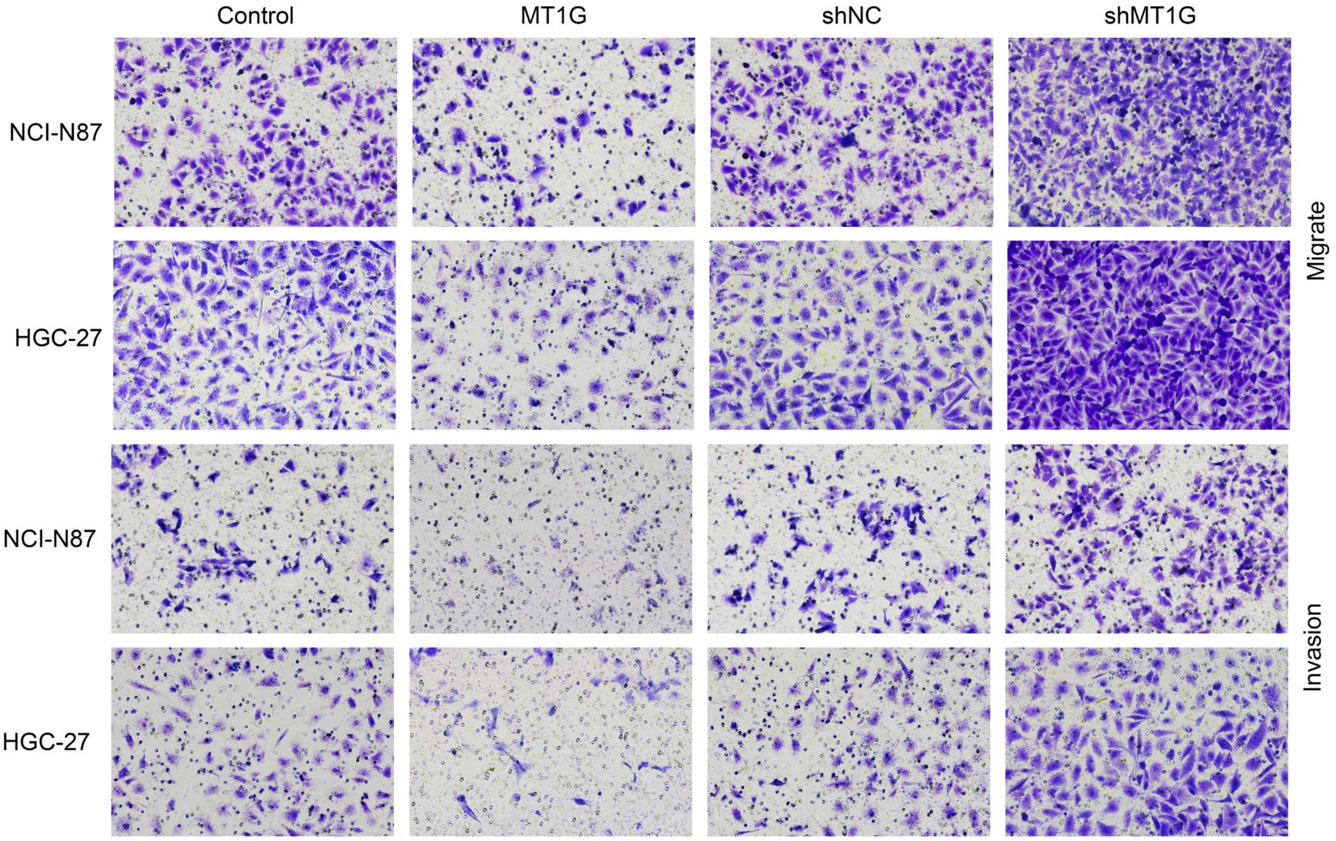

Figure S2 - MT1G negatively regulates cell migration and invasion. Transwell assay and transwell matrigel assay were used to measure cell migration and invasion in MT1G overexpression or knockdown NCI-N87 and HGC-27 cells.
